# Measuring health literacy in community agencies: a Bayesian study of the factor structure and measurement invariance of the health literacy questionnaire (HLQ)

**DOI:** 10.1186/s12913-016-1754-2

**Published:** 2016-09-22

**Authors:** Gerald R. Elsworth, Alison Beauchamp, Richard H. Osborne

**Affiliations:** Health Systems Improvement Unit, School of Health and Social Development, Deakin University, Geelong, Australia

**Keywords:** Health literacy, Health Literacy Questionnaire, HLQ, Bayesian structural equation modelling, BSEM, Measurement invariance

## Abstract

**Background:**

The development of the Health Literacy Questionnaire (HLQ), reported in 2013, attracted widespread international interest. While the original study samples were drawn from clinical and home-based aged-care settings, the HLQ was designed for the full range of healthcare contexts including community-based health promotion and support services. We report a follow-up study of the psychometric properties of the HLQ with respondents from a diverse range of community-based organisations with the principal goal of contributing to the development of a soundly validated evidence base for its use in community health settings.

**Methods:**

Data were provided by 813 clients of 8 community agencies in Victoria, Australia who were administered the HLQ during the needs assessment stage of the Ophelia project, a health literacy-based intervention. Most analyses were conducted using Bayesian structural equation modelling that enables rigorous analysis of data but with some relaxation of the restrictive requirements for zero cross-loadings and residual correlations of ‘classical’ confirmatory factor analysis. Scale homogeneity was investigated with one-factor models that allowed for the presence of small item residual correlations while discriminant validity was studied using the inter-factor correlations and factor loadings from a full 9-factor model with similar allowance for small residual correlations and cross-loadings. Measurement invariance was investigated scale-by-scale using a model that required strict invariance of item factor loadings, thresholds, residual variances and co-variances.

**Results:**

All HLQ scales were found to be homogenous with composite reliability ranging from 0.80 to 0.89. The factor structure of the HLQ was replicated and 6 of the 9 scales were found to exhibit clear-cut discriminant validity. With a small number of exceptions involving non-invariance of factor loadings, strict measurement invariance was established across the participating organisations and the gender, language background, age and educational level of respondents.

**Conclusions:**

The HLQ is highly reliable, even with only 4 to 6 items per scale. It provides unbiased mean estimates of group differences across key demographic indicators. While measuring relatively narrow constructs, the 9 dimensions are clearly separate and therefore provide fine-grained data on the multidimensional area of health literacy. These analyses provide researchers, program managers and policymakers with a range of robust evidence by which they can make judgements about the appropriate use of the HLQ for their community-based setting.

## Background

Researchers frequently present their findings from questionnaire development protocols and subsequent psychometric analyses as establishing that the questionnaire is ‘valid’. Such claims ignore long standing arguments that validity is inherent in the inferences derived from test scores, not a property of the tests themselves [1, 2]. Central to the process of providing evidence to potential users is validation of the interpretations derived from questionnaires in varying research contexts [3]. In the health sciences this needs to be an on-going and responsive process, requiring the generation of evidence to support emerging conclusions. “Validity is a property of inferences.” And not “…a property of designs or methods, for the same design may contribute to more or less valid inferences under different circumstances.” ([4], p. 34). It is therefore incumbent upon questionnaire developers to generate sound evidence for fellow researchers and evaluators that enables them to make judgments about the relevance and robustness of the questionnaire in different settings. This is important as the interpretation of scale scores may vary with new contexts and “…each interpretation of the scores needs to be validated…” by a “…program of research to support the …application of the tool in relation to an increasing range of interpretations…” ([5], p. 2).

The development and preliminary validation of the Health Literacy Questionnaire (HLQ) was reported in 2013 [6]. With the concept of health literacy being widely embraced in most regions of the world through the World Health Organisation (WHO), government agencies and researchers [7, 8], the HLQ’s constructs and items have been favourably received by stakeholders, resulting in uptake in over 30 countries and translation to over 15 languages. It is therefore timely to subject the HLQ to a new range of rigorous tests across different contexts of use.

The HLQ was generated from 91 items and 11 constructs derived from concept mapping. Psychometric analysis of data from the initial calibration sample identified 34 poorly performing or conceptually redundant items and they were removed resulting in 10 item clusters. These clusters were then tested in a replication sample and refined to yield 9 final scales comprising 44 items (5 scales on a 4-point ‘agree/disagree’ response continuum and 4 ‘cannot do/very easy’ scales on a 5-point continuum). A 9-factor CFA model using polychoric correlations and weighted least squares estimation (WLSMV) yielded a satisfactory fit considering the very restricted nature of the model where all potential cross-loadings and residual correlations were constrained to zero. Final scales were: 1. Feeling understood and supported by healthcare providers (4 items); 2. Having sufficient information to manage my health (4); 3. Actively managing my health (5); 4. Social support for health (5); 5. Appraisal of health information (5); 6. Ability to actively engage with healthcare providers (5); 7. Navigating the healthcare system (6); 8. Ability to find good health information (5); and 9. Understand health information well enough to know what to do (5). Composite reliability of the HLQ scales ranged from 0.77 (5. Appraisal of health information) to 0.90 (8. Ability to actively engage with healthcare providers). Correlations between factors were reported to show clear discrimination between the ‘disagree/agree’ scales (range 0.43 to 0.78). Less discrimination was, however, evident for the ‘cannot do/very easy’ scales (range 0.83 to 0.93). This weaker discrimination was interpreted as “suggesting higher order factors may be present, including a general capability to interact positively and effectively with the healthcare system.” [6]. More information about the HLQ is available on a website (https://www.ophelia.net.au). Current information on the availability of translations into languages other than English can be obtained from the authors.

While the calibration and validation samples of respondents that supported the development of the HLQ were largely drawn from clinical and home-based aged-care settings, the questionnaire was designed for use in the full range of healthcare contexts, including community-based health promotion and support services. This paper is thus designed to investigate the potential of the HLQ to provide valid inferences when used across a broader range of community-based organisations. The principal goal of the paper is to contribute to the development of a soundly validated evidence base for its use in community health settings. The aims of the paper are to: (a) replicate the homogeneity and reliability of the individual HLQ scales in a new sample of respondents from a diverse range of community health settings; (b) replicate the 9-factor structure of the HLQ in this sample; (c) investigate further the discriminant validity of the HLQ scales; and (d) establish the measurement invariance of the scales across different types of agencies and the gender, language background, age and educational level of respondents.

## Methods

### Data sources

Data from the administration of Version 1 of the HLQ in the initial phase of a large multi-centre service improvement trial, the Ophelia (OPtimising HEalth LIteracy and Access) project [9] were used. The data were provided by 813 clients of 8 diverse community-based agencies in Victoria, Australia who were administered the HLQ during the needs assessment stage of the project. The settings and questionnaire respondents who were recruited for the study are described in detail elsewhere [9, 10]. Briefly, 8 organisations providing Home and Community Care (HACC) services, Hospital Admission Risk Programs (HARP) or community nursing and other chronic disease services from 4 of 8 Department of Health (now Department of Health and Human Services) regions in Victoria were invited to participate in the Ophelia project through an expression of interest process. The respondents comprised people attending one of these 8 participating organisations. Each organisation selected a target group of clients based on a service-provision priority. The majority of the participants were expected to have a chronic health condition although this was not a pre-requisite for inclusion. Trained staff from each organisation collected data from a representative sample of clients within their target group using consecutive methods of recruitment where feasible and employing various strategies for recruiting clients who are traditionally ‘harder to reach’. Selection criteria required that participants should be cognitively able to provide informed consent to participate, and be over the age of 18 years.

Numbers in the selected organisations available for data analysis were:

1. A Melbourne metropolitan municipal community service = 102 cases;
2. A rural coastal community health service = 70;
3. A regional city case management service for chronic and complex clients = 132;
4. A Melbourne metropolitan community health service = 90;
5. A Melbourne outer metropolitan community health service = 108;
6. A Melbourne outer metropolitan municipal community service = 97;
7. A regional community health service = 99;
8. A Melbourne metropolitan domiciliary nursing service = 115.

### Data analysis

Analyses were conducted with Mplus Version 7.4. Composite scale reliability was calculated using the Mplus code developed by Raykov [11–13] with robust maximum likelihood estimation (MLR). All other analyses used Bayesian approaches; specifically Bayesian structural equation modelling (BSEM) [14–16] and Bayesian alignment analysis [17]. The Bayesian approaches to CFA and the investigation of measurement invariance are outlined below. Measurement invariance was investigated across gender, age (approximate quintiles), education (a five-category classification ranging from ‘Primary school or less’ to ‘University’), language spoken at home (English, yes/no), and the organisation where the respondent was a client.

### Composite scale reliability and homogeneity

We define ‘reliability’ in the classical sense of the ratio of the true score variance to the observed variance of a test or other measuring instrument as estimated by the composite reliability coefficient [11–13]. If the proportion of true score variance reaches an acceptable level when a test is used for a particular purpose in a given sample of respondents it is assumed that it will provide repeatable parameter estimates when used in similar ways in similar populations of respondents. Reliability can, however, be too high, and when very high is often a symptom of the test consisting of conceptually or linguistically redundant items, and thus one not measuring a construct of sufficient breadth to be practically or theoretically useful [18]. A threshold value for composite reliability that is frequently regarded as satisfactory is 0.8 and that value was used as a benchmark in this study [13].

Homogeneity (i.e. unidimensionality), as distinct from reliability, is defined as the existence of a single latent variable underlying each hypothesised item cluster [19, 20] and thus as a properly specified independent clusters measurement model (ICM) having acceptable fit to the data [11, 13, 21]. Scale homogeneity was investigated using single-factor BSEM models.

### Discriminant validity

Comparison of the average variance extracted (AVE) and the variance shared with other scales provides evidence of discriminant validity [22, 23]. The criteria for discriminant validity are well summarised by Farrell ([23] p. 324): “Discriminant validity is the extent to which latent variable A discriminates from other latent variables (e.g., B, C, D). Discriminant validity means that a latent variable is able to account for more variance in the observed variables associated with it than a) measurement error or similar external, unmeasured influences; or b) other constructs within the conceptual framework. If this is not the case, then the validity of the individual indicators and of the construct is questionable (Fornell and Larcker, 1981).” The first criterion can be tested by examining the absolute size of the AVE; if it is >0.5 then >50 % of the average variance of the items that comprise the scale is accounted for by the latent variable (factor) while, by definition, <50 % is associated with other sources: measurement error, unique item variance and, in a multi-factor model with cross-loadings allowed, the contribution of other factors to the variance of the item. The second criterion can be tested by comparing the AVE of each factor of each pair with the inter-factor correlation between that pair of factors; estimates of the AVE of both factors are required to be greater than the shared variance between them ([23] p. 325).

### Bayesian structural equation modelling

BSEM is a specific application of Bayesian statistical analysis to factor analysis and structural equation modelling [14]. Bayesian analysis views the parameters of a structural equation model as variables, compared with the more typical ‘frequentist’ approaches where the model parameters are viewed as constants. The distribution of the parameters in a Bayesian analysis is referred to as a *prior*. Priors can be either *diffuse* (non-informative) or *informative* ([14], p. 314). Informative priors, derived from either partial knowledge of the parameter distributions (e.g. from similar studies, pilot studies etc.) or on the basis of theory are at the heart of typical Bayesian analyses. Diffuse priors can also be used, however, in which case a Bayesian analysis would be anticipated to give very similar results to a maximum likelihood (ML) analysis in a large sample. The Bayesian analysis incorporates the information in the prior along with information from the data to generate a *posterior* distribution. When informative priors are used, the resulting solution is derived from a compromise between a likelihood function computed from the data and the prior. The larger the variance of the prior, the more weight the actual data have in determining the resulting posterior distribution.

Crucially, for the present study, BSEM enables small variance informative priors to be established for all major parameters in a SEM model. In a CFA with more than one latent variable, these parameters include possible cross-loadings and residual correlations. In a conventional CFA using a frequentist approach (with either maximum likelihood or weighted least-squares estimation) these parameters are typically set to exactly zero although modification indices may be used to suggest individual cross-loadings or residual correlations that can be freely estimated. In larger multi-factor models with subjective self-report items or scales it is frequently the case that quite a large number of model modifications will be required to achieve an acceptable model fit [14, 24, 25]. Fitting model modifications one at a time (so long as the inclusion is ‘supported by theory’) as is typically recommended (e.g. [26]) may, however, not necessarily lead to a single solution depending on the sequence of individual decisions made, and if all available fixed parameters were to be freely estimated the model would be unidentified. BSEM offers a solution to this problem in the possibility that small deviations from the rigorous requirement of fixed strictly zero cross-loadings and/or residual correlations can be incorporated into the model using small variance priors (‘wiggle room’ [15]) thus potentially enabling achievement of good model fit and consequent unbiased estimation of model parameters.

Asparouhov and Muthén also proposed and programmed in Mplus a “new method for multiple-group … CFA” referred to as the “alignment method” or “alignment optimization” ([17], p. 2). The method is designed to enable the unbiased estimation of the factor means of multiple groups “without requiring exact measurement invariance”. The alignment method is based on the configural invariance model. After fitting the configral model, the alignment optimization approach then uses a simplicity criterion (analogous to that used in factor rotation to simple structure) to locate the most optimal pattern of measurement invariance across groups. The chosen simplicity criterion is a loss function that is minimized when there are many approximately invariant measurement parameters (item factor loadings and item intercepts) and a small number of large non-invariant measurement parameters. In addition to providing information about the significantly different factor means based on this optimized invariance pattern the program output provides information on the fit of the configural model and, importantly for this study, the pattern of optimized invariance/non-invariance of each measured variable across each group. The alignment method is available for use with both maximum likelihood and Bayesian estimation.

#### Fit indices used in Bayesian structural equation modelling

BSEM uses a different approach to assessing model fit in comparison to frequentist approaches, although both are based on the calculation of a chi-square likelihood statistic. Model fit is assessed using a procedure called ‘posterior predictive checking’ that generates a ‘Posterior Predictive P value’ (PPP value) which, in a very well fitting model, is expected to be close to 0.5 while a low value, approaching 0.0, indicates poor fit. Additionally a fit statistic for the difference between the observed and replicated Chi-square values derived from the Monte Carlo Markov Chain (MCMC) fitting algorithm is calculated with 95 % confidence intervals (CIs). Symmetrical lower and upper 95 % CIs centred on zero align with a PPP value of 0.5 in suggesting excellent fit while a lower 95 % CI that is positive accords with a PPP value approaching 0.0 and indicates poor fit. “An excellent-fitting model is expected to have a PPP value around 0.5 and an *f* statistic difference of zero falling close to the middle of the confidence interval.” Also the usual approach to statistical significance of “…using posterior predictive p value values of .10, .05, or .01 appears reasonable.” ([14], p. 315). Hence a PPP value of, for e.g., >0.05 would be interpreted as suggesting that the discrepancy between the postulated factor model and the data is not statistically significant and thus the model is an acceptable fit to the data. These two indicators of model fit are used in both CFA and invariance analyses reported below.

### Population heterogeneity and measurement invariance

Population heterogeneity refers to the possibility that the parameters of statistical models may vary across different sub-groups within a broadly defined population. These sub-groups can be either observed or unobserved. When the possible sources of heterogeneity are observed the population can be split *a priori* into the observed sub-groups and the data analysed with a variety of methods developed for modelling data from multiple groups [27, 28]. When the modelling is based on factor analysis the parameters that are of primary interest are typically the factor loadings and item intercepts although the item residuals and covariances and, in multi-factor models, the factor covariances might also be considered [29].

Observed population heterogeneity can be planned or passively observed. When planned, the population sub-groups might be formed from experimental manipulation or generated by a multi-group sampling design where, for example, data are gathered purposefully from organizations offering tailored services to contrasting client groups (as in the present study) or oversampling strategies are employed to ensure representation of specific population groups with special needs. Given that the equivalence (invariance) of specific model parameters can be demonstrated across the groups of interest, valid comparisons of these parameters (e.g. means, variances, correlations, regression/path coefficients etc.) across the groups can be made.

From a measurement perspective, the use of multiple-item composite scales for group comparisons is critically dependent on the demonstration that: (a) the same factor structure underpins the items in all groups of interest (configural invariance); (b) the factor loadings are equivalent across groups (metric invariance); and (c) item intercepts or thresholds in the case of ordered categorical variables are also equivalent across the groups (scalar invariance) (see e.g. [29, 30]). While recent commentary throws some doubt on the necessity for strict metric invariance for group comparisons [31, 32], the need for scalar invariance is strongly emphasized, particularly if composite (summed or averaged) scores are used [32] . Typically, however, these three aspects of measurement invariance are viewed as a hierarchy; in particular, metric and scalar invariance cannot be supported unless configural invariance is demonstrated (see e.g. [30] p. 382).

Factorial invariance of the HLQ was investigated following recent advocacy for a refocus of the usual statistical approach by Raykov and colleagues [33]. Typically, invariance is investigated by fixing factor loadings and item intercepts (or thresholds) to equality across groups or time in a hierarchical manner [30, 34]. But it is argued that the structural equation models used in this approach have significant limitations, and, in general, don’t provide a complete and unconditional statistical assessment of metric or scalar invariance [35]. Hence it is recommended that, at present, metric and scalar invariance be investigated using only an unconditional model (i.e. a model in which no factor loadings or intercepts/thresholds are fixed to 1.0) with a complete set of equality constraints for metric and scalar invariance but minimum constraints necessary for model identification. The Mplus default for this kind of analysis with categorical variables and Bayesian estimation was used which constrains all factor loadings and item thresholds to be equal across groups and, in addition, fixes all factor variances to 1.0 and the mean of the last variable to zero for identification. In addition, while item residual covariances were allowed to deviate a little from zero using small variance priors, they were constrained to be equal across groups. This resulted in very strict invariance models; all factor loadings and variances were constrained to be equal together with all item thresholds and residual covariances. Only factor means, aside from the mean of the last group were free to vary. (As the Mplus default for these analyses was the Delta parameterization, in which the item variances are not part of the model, the scale factors, with the exception of that for a reference item, were free to vary. In the Delta parameterization the residual item variances can be retrieved from the R-squared estimates for the items, which, in the fully constrained invariance model, are equal across groups.)

Additionally, the alignment method, described briefly above, was used as a follow-up analysis to provide more specific information on the non-invariant model parameter (item factor loading or intercept) and group when non-invariance of a specific HLQ item was evident in the BSEM analyses. Fit of the configural models was maximised by allowing a small number of correlated residuals (maximum 4) based on estimates from BSEM analyses of the single-factor measurement models without a grouping variable. As the data from the HLQ frequently show strongly non-normal distributions, the Bayesian alignment approach was used while the ‘FREE’ alignment option that provides least biased estimates in analyses involving more than 2 groups was employed as appropriate.

## Results

### Reliability and homogeneity of the HLQ scales

Estimates of the composite reliability of the HLQ scales in the Ophelia sample (together with Cronbach’s alpha for possible comparison with studies of other scales) are shown on the last column of Table 1. All estimates of reliability are ≥0.8 but <0.9 suggesting good reliability without item redundancy.

**Table 1:** Bayesian Modelling and Reliability of HLQ Single Scales

Scale	Model	PPP Value	95 % Cis for the difference between observed and replicated chi-square values	Standardised factor loadings from ‘Wiggle Room’ analysis	Range of residual correlations in ‘Wiggle Room’ analysis	Composite reliability (α in parentheses)
1. Feeling understood and supported by healthcare providers	No residual correlations	0.35	−12.45–18.83	P1Q2 = 0.80; P1Q8 = 0.90; P1Q17 = 0.76; P1Q22 = 0.90.	−0.12 to 0.01	0.86 (0.86)
	Residual correlations estimated	0.51	−14.98–14.02			
2. Having sufficient information to manage my health	No residual correlations	0.33	−11.95–21.30	P1Q1 = 0.67; P1Q10 = 0.82; P1Q14 = 0.89; P1Q23 = 0.91.	−0.09 to 0.15	0.85 (0.84)
	Residual correlations estimated	0.52	−15.12–16.15			
3. Actively managing my health	No residual correlations	0.34	−16.17–24.69	P1Q3 = 0.69; P1Q5 = 0.79; P1Q11 = 0.84; P1Q18 = 0.81; P1Q86 = 0.86.	−0.13 to 0.17	0.85 (0.85)
	Residual correlations estimated	0.52	−17.79–14.79			
4. Social support for health	No residual correlations	0.00	13.41–64.96	P1Q3 = 0.68; P1Q5 = 0.66; P1Q11 = 0.94; P1Q15 = 0.55; P1Q19 = 0.81.	−0.17 to 0.32 (0.32 = P1Q15/P1Q19)	0.80 (0.80)
	Residual correlations estimated	0.51	−17.18–16.38			
5. Appraisal of health information	No residual correlations	0.18	−11.55–30.15	P1Q4 = 0.72; P1Q7 = 0.74; P1Q12 = 0.82; P1Q16 = 0.67; P1Q20 = 0.62.	−0.13 to 0.17	0.81 (0.81)
	Residual correlations estimated	0.49	−16.92–18.23			
6. Ability to actively engage with healthcare providers	No residual correlations	0.04	−2.12–42.10	P2Q2 = 0.81; P2Q4 = 0.85; P2Q7 = 0.83; P2Q15 = 0.85; P2Q20 = 0.87.	−0.23 to 0.19	0.89 (0.89)
	Residual correlations estimated	0.52	−18.00–17.28			
7. Navigating the healthcare system	No residual correlations	0.02	1.79–49.19	P2Q1 = 0.76; P2Q8 = 0.77; P2Q11 = 0.82; P2Q13 = 0.91; P2Q16 = 0.73; P2Q19 = 0.66	−0.09 to 0.24	0.87 (0.87)
	Residual correlations estimated	0.51	−20.40─20.45			
8. Ability to find good health information	No residual correlations	0.01	−3.89–47.04	P2Q3 = 0.80; P2Q6 = 0.79; P2Q10 = 0.84; P2Q14 = 0.66; P2Q18 = 0.72	−0.19 to 0.14	0.84 (0.84)
	Residual correlations estimated	0.50	−17.82–17.67			
9. Understanding health information well enough to know what to do	No residual correlations	0.00	18.17–68.47	P2Q5 = 0.82; P2Q9 = 0.60; P2Q12 = 0.89; P2Q17 = 0.81; P2Q21 = 0.74.	−0.16 to 0.35 (0.35 = P2Q9/P2Q21)	0.85 (0.83)
	Residual correlations estimated	0.50	−17.98–18.52			

Bayesian fit statistics for 9 single factor models that either: (a) fixed residual correlations to zero, or (b) allowed residual correlations to be estimated with a small variance prior to give a 95 % probability that the correlations were within the range of ±0.2 ([14], p. 317) are also shown in Table 1 together with the factor loadings and the range of residual correlations resulting from the ‘wiggle room’ analysis.

The fit of the single factor analyses without ‘wiggle room’ was satisfactory for 4 scales (1. Feeling understood and supported by healthcare providers; 2. Having sufficient information to manage my health; 3. Actively managing my health; and 5. Appraisal of health information). For the other 5 scales either the PPP value was <0.05 or the lower CI for the difference between the observed and replicated Chi-square values was positive, or both. However, when a small variance prior was used for the residual correlations all models were an excellent fit on both criteria. Furthermore only two residual correlations were found to be >0.3, that is, in only two instances was the within-construct overlap between the items after accounting for the single latent variable >10 %. All factor loadings, with one exception (0.55) were 0.6 or higher, suggesting that almost all items were quite strongly associated with the hypothesised construct.

### Replicating the nine-factor structure of the HLQ

To investigate whether the previously established factor structure of the HLQ was replicated, a 9-factor model was fitted to the data. Bayesian estimation was used with small variance priors for both cross-loadings and residual correlations such that small deviations of both sets of parameters from precisely zero were allowed. The variance of the priors for the cross-loadings was initially set at 0.02 such that there was a 95 % probability that the cross-loadings would be within the range ±0.28 ([14], p 317, Table 2). Similarly, the variance for the residual correlations was set to give a 95 % probability that the correlations were within the range of ±0.2. This model fitted the data very well, but as one important aim of the analysis was to locate the best estimates of the factor loadings and inter-factor correlations, the priors for the cross-loadings were systematically studied across values that would yield a 95 % probability that the cross-loadings were within ranges that varied from ± 0.20 to ± 0.34. A prior variance of 0.021, slightly larger than that originally tested, was found by this process to give the best fit (PPP = 0.514; 95 % CIs for the difference between the observed and replicated Chi-square values = −131.8–128.8). Table 2 shows the pattern of statistically significant target and non-target factor loadings from this analysis. Correlations between the factors are shown in the lower-left part of the matrix in Table 3.

**Table 2:** Factor Loadings – Nine-factor Model of the HLQ

Item	1. Understood	2. Sufficient information	3. Active management	4. Social support	5. Appraisal	6. Active engagement	7. Navigate	8. Good information	9. Understand information
P1Q2	0.83								
P1Q8	0.93								
P1Q17	0.53	0.17							−0.10
P1Q22	0.86								
P1Q1		0.39							
P1Q10		0.65							
P1Q14		0.92							
P1Q23		0.88							
P1Q6			0.72						
P1Q9			0.78						
P1Q13			0.92						
P1Q18			0.81						
P1Q21			0.96						
P1Q3	0.18			0.55					
P1Q5				0.68					
P1Q11				0.93					
P1Q15				0.70					
P1Q19				1.02					
P1Q4					0.76				
P1Q7					0.78				
P1Q12					0.86				
P1Q16		0.19			0.47	0.09	0.09	0.09	
P1Q20	0.15				0.48				
P2Q2						0.71			
P2Q4						0.78			
P2Q7						0.75			
P2Q15						0.77			
P2Q20						0.83			
P2Q1							0.68		
P2Q8							0.88		−0.13
P2Q11							0.89		
P2Q13							0.78		
P2Q16							0.57		
P2Q19	−0.16						0.62		
P2Q3								0.86	
P2Q6					0.14			0.84	
P2Q10								0.92	
P2Q14						0.22		0.40	0.29
P2Q18	−0.16						0.22	0.41	0.20
P2Q5						0.14		0.14	0.63
P2Q9						0.15	0.17	0.15	0.22
P2Q12							0.08		0.83
P2Q17									0.76
P2Q21						0.25		0.25	0.36

Note: all statistically significant (pr. <0.05) factor loadings shown

Model Fit: Posterior Predictive P-Value = 0.514; 95 % Confidence Interval for the Difference between Observed and Replicated Chi-Square Values = −131.838–128.768

**Table 3:** Inter-factor Correlations (below diagonal) Average Variance Extracted (diagonal) and Shared Variance Estimates (above diagonal) for the nine HLQ scales

	1. Understood	2. Sufficient information	3. Active management	4. Social support	5. Appraisal	6. Active engagement	7. Navigate	8. Good information	9. Understand information
1. Feeling understood and supported by healthcare providers	***0.63***	0.45	0.27	0.51	0.12	0.41	0.30	0.16	0.02
2. Having sufficient information to manage my health	0.67*	***0.55***	0.38	0.51	0.19	0.42	0.39	0.35	0.03
3. Actively managing my health	0.52*	0.62*	***0.71***	0.38	0.43	0.20	0.19	0.25	0.11
4. Social support for health	0.71*	0.72*	0.62*	***0.63***	0.18	0.44	0.41	0.30	0.05
5. Appraisal of health information	0.34*	0.44*	0.66*	0.42*	***0.47***	0.08	0.09	0.26	0.08
6. Ability of actively engage with healthcare providers	0.64*	0.65*	0.45*	0.66*	0.27	***0.59***	0.79	0.65	0.19
7. Navigating the health system	0.55*	0.62*	0.44*	0.64*	0.30	0.89*	***0.56***	0.71	0.27
8. Ability to find good health information	0.40*	0.59*	0.50*	0.55*	0.51*	0.80*	0.85*	***0.53***	0.30
9. Understanding health information well enough to know what to do	0.15	0.16	0.34*	0.21	0.28	0.43*	0.52*	0.54*	***0.37***

Note: (i) Statistically significant (pr < 0.05) correlations are asterisked;

(ii) Average variance extracted (AVE) by each latent variable is in bold italics (on the diagonal):

(iii) Latent variable shared variance estimates that exceeded the AVE of either or both variables underlined

The results suggest that all items in two of the ‘disagree/agree’ scales and one ‘cannot do/very easy’ scale are strictly uni-factorial, fulfilling McDonald’s criterion for an ICM that “in a *confirmatory factor/item response model*, … each variable loads on just one factor …” ([20] p. 460, italics in original). The other four ‘disagree/agree’ scales had varying numbers of items that showed some multi-factoriality in that one or more had statistically significant non-target loadings; all non-target loadings were, however small (less than 0.2). Item P1Q16 ‘I know how to find out if … health information … is right or not’ appears to have the most complex factor structure, being significantly associated with four factors other than its hypothesised target construct. The strongest of these ‘non-target’ loadings was on Scale 2: Having sufficient information to manage my health, a readily understandable association. Three of the four ‘cannot do/very easy’ scales had a number of items that showed evidence of multi-factoriality, however. While one scale (6. Ability to actively engage with healthcare providers) had a pattern of strong target loadings and two statistically significant but small non-target loadings, two (Scales 8. Ability to find good health information and 9. Understanding health information well enough to know what to do) had two items with relatively weak target loadings and a number of items that revealed a rather stronger pattern of multi-factoriality than seen in other items. It is important to note, however, that all target loadings on these, and all other, scales were higher than any statistically significant non-target loading.

Correlations between the factors ranged from 0.15 to 0.89 suggesting satisfactory discrimination between the nine scales of the HLQ with the possible exception of Scales 6, 7 and 8 where the inter-factor correlations are 0.80, 0.85 and 0.89. The strongest of these inter-factor associations is between Scale 6 ‘Ability to actively engage with healthcare providers’ and Scale 7 ‘Navigating the healthcare system’.

### Convergent and discriminant validity

Along with the inter-factor correlations, Table 3 also shows the AVE and the shared variance between each factor and the other factors in the 9-factor model. It can be seen that the AVE for seven of the nine HLQ scales was >0.5 whereas the AVE for two (Scales 5 and 9) was <0.5. In interpreting this result, it should be kept in mind that these estimates of the AVE were derived from a multi-factor model with inter-factor correlations and cross-loadings allowed such that variance associated with other factors will potentially explain some of the variance in the items in the scale. This is particularly likely in Scale 9 where two items in particular appear to be quite strongly associated with other constructs, thus resulting in the apparent differences between the factor loadings shown in Table 1 and those in Table 2. Table 3 also shows that the shared variance between all pairs of factors in the cluster of Scales 6, 7 and 8 is greater than the AVE of the factors. In contrast, the AVEs calculated from the one-factor models were, respectively, 0.71, 0.68, 0.64, 0.55, 0.51, 0.71, 0.60, 0.58 and 0.61, all above the threshold of 0.5. Fornell and Larker’s second criterion was, however, also not satisfied for scales 6, 7 and 8 when this calculation of the AVE was used. This suggests that there may be insufficient discriminant validity between these three scales.

### Measurement invariance of the HLQ scales

Bayesian fit statistics resulting from fitting the single-factor models across contrasting groups representing gender, age, education level, language spoken at home and the organisation where the respondent was a client are summarised in Table 4. The strict invariance model was a satisfactory fit across all analyses involving all ‘disagree/agree’ scales of the HLQ. It was also a satisfactory fit across all ‘cannot do/very easy’ scales of the questionnaire when the respondents were classified by gender. All ‘cannot do/very easy’ scales were, however, non-invariant when clients were classified by their organisation (albeit, for Scale 6 (Ability to actively engage with healthcare providers), while model fit was not satisfactory overall, it was satisfactory in each group individually). Additionally Scale 7 (Navigating the healthcare system) was non-invariant across age and education level; Scale 8 (Ability to find good health information) was non-invariant across education level and (marginally) home language; while Scale 9 (Understanding health information well enough to know what to do) was non-invariant across education level.

**Table 4:** Bayesian Model Fit Statistics for Invariance Analyses

Scale	Grouping Variable	PPP Value	95 % CIs for the Difference between Observed and Replicated Chi-square values	Range of PPP Values Across Individual Groups
1. Feeling understood and supported by healthcare providers	Gender	0.54	−20.37–19.54	0.51–0.52
	Age	0.29	−23.83–43.37	0.31–0.49
	Education	0.26	−21.77–46.97	0.19–0.49
	Home Language	0.26	−16.47–28.74	0.02–0.49
	Organisation	0.14	−14.24─74.27	0.20─0.43
Comment: Strict invariance models were a satisfactory fit across all classifications and across all specific groups within these classifications.				
2. Having sufficient information to manage my health	Gender	0.37	−18.41–22.12	0.40–0.41
	Age	0.38	−28.33–38.36	0.35–0.50
	Education	0.30	−24.53─43.38	0.17–0.57
	Home Language	0.53	−22.80–18.07	0.49–0.56
	Organisation	0.12	−16.82–68.33	0.07–0.60
Comment: Strict invariance models were a satisfactory fit across all classifications and across all specific groups within these classifications				
3. Actively managing my health	Gender	0.23	−15.20–34.12	0.03–0.32
	Age	0.09	−7.73–70.08	0.14–0.36
	Education	0.22	−26.39–58.54	0.24–0.54
	Home Language	0.46	−23.82–26.67	0.45–0.50
	Organisation	0.34	−30.59–66.52	0.26–0.55
Comment: Strict invariance models were a satisfactory fit across all classifications and across all specific groups within these classifications.				
4. Social support for health	Gender	0.28	−17.35–32.76	0.39–0.35
	Age	0.14	−18.41–60.05	0.22–0.45
	Education	0.42	−33.56–44.89	0.34–0.65
	Home Language	0.36	−21.46–25.62	0.47–0.35
	Organisation	0.12	−20.47–79.84	0.10–0.58
Comment: Strict invariance models were a satisfactory fit across all classifications and across all specific groups within these classifications.				
5. Appraisal of health information	Gender	0.50	−24.74–23.86	0.47–0.54
	Age	0.61	−41.51–29.24	0.41–0.65
	Education	0.48	−36.51–39.77	0.32–0.61
	Home Language	0.11	−10.01–43.82	0.08–0.47
	Organisation	0.12	−25.04–85.85	0.15–0.42
Comment: Strict invariance models were a satisfactory fit across all classifications and across all specific groups within these classifications.				
6. Ability to actively engage with healthcare providers	Gender	0.15	−11.89–40.68	0.20–0.31
	Age	0.11	−14.81–70.04	0.12–0.48
	Education	0.09	−12.96–67.97	0.10–0.41
	Home Language	0.18	−13.98–38.54	0.12–0.47
	Organisation	0.02	2.52–107.55	0.07–0.52
Comment: Overall test of invariance across organisations marginally significant (indicating unsatisfactory fit) but fit was satisfactory in all groups separately. Satisfactory fit across all other classifications. Follow-up alignment analysis indicated that the loading of one item (‘Ask healthcare providers questions …’) was non-invariant in Organisation 1, being consistently higher in Organisation 1 than in other organisations.				
7. Navigating the healthcare system	Gender	0.12	−11.90–49.41	0.16–0.28 -
	Age	0.01	15.64–111.02	0.02–0.41
	Education	0.02	4.90–99.30	0.12–0.26
	Home Language	0.13	−12.53–48.144	0.10–0.398
	Organisation	0.01	24.17–144.22	0.02–0.33
Comment: Fit not satisfactory across organisation, education and age groups. Follow-up alignment analyses showed: (a) that the loading of one item (‘Work out what the best care is …’) was non-invariant in Organisation 6, being consistently higher in this organisation compared with the other organisations. Alignment analysis suggested full metric and scalar invariance across education and age groups.				
8. Ability to find good health information	Gender	0.34	−21.52–27.64	0.39–0.45
	Age	0.12	−15.77–62.40	0.14–0.52
	Education	0.00	21.38 - 104.91	0.00–0.35
	Home Language	0.04	−3.82–52.07	0.03–0.30
	Organisation	0.00	21.78–125.37	0.01–0.57
Comment: Fit not satisfactory across organisation, education and, marginally, home language groups. Follow-up alignment analyses showed that: (a) the loading of one item (‘Find information about health …’) was non-invariant in Organisation 4, being consistently lower in this organisation compared with all other organisations; and (b) the loading of one item (‘Get health information … you understand’) was non-invariant across the groups classified according to the language spoken at home, being higher in the group who spoke a language other than English at home. Alignment analysis suggested full metric and scalar invariance across the education groups.				
9. Understanding health information well enough to know what to do	Gender	0.31	−19.56–31.24	0.31–0.44
	Age	0.24	−27.09–50.95	0.25–0.64
	Education	0.022	2.15 - 79.32	0.10–0.38
	Home Language	0.16	−13.47–42.17	0.36–0.16
	Organisation	0.00	26.61–132.36	0.01–0.72
Comment: Fit not satisfactory across organisation and education level. Follow-up analysis showed that: (a) the loadings of two items, both of which referred to understanding information from healthcare providers, were non-invariant in Organisation 8 being consistently lower in Organisation 8 that in all other organisations; and (b) the loading of one item (‘Read and understand … medication labels’ was non-invariant in Education Group 2, being higher than in Groups 3, 4 and 5, but lower than in Group 1, suggesting that this item is most salient in the ‘Understanding health information well enough to know what to do’ factor in the groups with less formal education.				

Each of these indications of non-invariance was followed up by an alignment optimization analysis to locate more specifically if the non-invariance was metric or scalar along with the specific item and group(s) it was associated with. The results of these analyses are summarised briefly in the appropriate ‘Comment’ section of Table 4. Overall, all of the approximate non-invariance detected by these analyses was associated with factor loadings (i.e. metric non-invariance) rather than item intercepts (scalar non-invariance); also most of the metric non-invariance detected was associated with one or at most two items when the data were classified by the client’s organisation. Two other specific sources of metric non-invariance were the language spoken at home and the educational level of the respondent.

## Discussion

These analyses of the HLQ in disparate settings using, largely, the Bayesian approach to structural equation modelling have provided a rigorous assessment of its psychometric properties in a sample of clients of a diverse group of community agencies. The principal goal of the paper was to contribute to the development of a sound evidence base for the valid use of the HLQ in community health settings. This goal was to be addressed by replicating the homogeneity, reliability and 9-factor structure of the HLQ scales for use in this setting, investigating further the discriminant validity of the scales, and establishing their measurement invariance across a diverse range of organisations and salient sociodemographic variables. These specific aims are addressed in turn in the following paragraphs.

When a small variance Bayesian prior was used to allow modest correlations among the item residuals, single factor CFA models for all HLQ scales were found to fit the data very well, thus establishing a satisfactory level of scale homogeneity. Additionally, the composite reliability of all scales, with only between 4 and 6 items, was >0.8.

A 9-factor model using small variance Bayesian priors for both cross-loadings and residual correlations similarly fitted the data very well thus replicating the hypothesised factor structure. All statistically significant cross-loadings were ≤0.25 and lower that their associated target loading. This model was also used to investigate the discriminant validity of the scales. Comparing the inter-factor correlations of each pair of HLQ scales to the average variance extracted by each scale in the pair clearly established the discriminant validity of 6 of the HLQ scales: all ‘agree/disagree’ scales and (even though its AVE was relatively low) Scale 9 from the ‘cannot do/very easy’ group (Understanding health information well enough to know what to do). The three other ‘cannot do/very easy’ scales did not show sufficient discriminant validity to establish a clear psychometric distinction between the constructs, however. The suggestion was made in the HLQ development paper that a higher-order factor may explain the relatively higher correlations between some of the ’cannot do/very easy’ scales [6]. The cluster of scales with high inter-factor correlations in the present analysis supports this view. All items in Scales 6, 7 and 8 broadly connote a proactive approach to interactions with the healthcare system in relation to contact and collaboration with healthcare providers, navigating the system and obtaining information.

Notwithstanding these psychometric indications of insufficient discriminant validity of some scales, extensive field work, clinical interactions, and epidemiological work continue to support the application of the scales as independent indicators of a broad range of personal and social dimensions of health literacy. A recent epidemiological report showed different patterns of association of the HLQ scales with a number of important socio-demographic variables [10], while studies across three groups of South African residents living in an informal settlement outside Cape Town indicated somewhat different scale scores, and the items and scales were very meaningful to local clinicians and researchers [36]. Additionally, numerous clinical consultations using each scale employing the Ophelia process [9] indicate the content of individual scales provides separate and useful information.

Overall, the data from this study and concurrent field work clearly show that HLQ scales measure different concepts. The inter-factor correlations indicate that some scales are highly correlated, namely 6, 7 and 8. This suggests that either a higher order factor or underlying causal connections in specific population groups might be present. In some settings high correlations can mean construct overlap and that the items might be best combined. This is unlikely to be the case here for several reasons: (a) the item content is underpinned by the results of concept-mapping that clearly differentiated distinct constructs; (b) the scales tend to be associated differently with important exogenous variables; and (c) clinical and health promotion groups have carefully considered potential interventions related to these scales and quite different interventions have been proposed [9]. Factor analysis does not necessarily fully resolve issues associated with the conceptual structure of psychological measures [37]. The logic of construct validation requires consideration of both the internal structure of a measuring instrument and its relationships with theoretically relevant exogenous variables (its ‘nomological network’ [38]). Differentiation both *within* the structure of a multi-scale measure and *between* the individual scales of the multi-scale measure and theoretically salient variables in the nomological network provides accumulating evidence to support discriminant validity in varying contexts. The wide variety of studies using the HLQ that are underway will continue to expand the evidence base for the discriminant validity of the nine scales in specific contexts.

Measurement invariance of the HLQ was investigated one scale at a time. A very strict measurement invariance model was studied in which all factor loadings, item intercepts, factor variances and item residual covariances were fixed to equality across groups and which also resulted in equality of item residual variances. When non-invariance was evident a follow-up alignment optimisation analysis was performed to establish more fully the nature of the non-invariance. All ‘disagree/agree’ scales were found to be fully invariant across the gender, age, educational level and the language background of the respondents as well as the organisations in which they were clients. Measurement invariance of Scales 6, 7, 8 and 9 was less well established. All four of these ‘cannot do/very easy’ scales were invariant across gender. Scale 6 (Ability to actively engage with healthcare providers) was, however, not fully invariant across organisation, Scales 7 and 9 (Navigating the healthcare system, Understanding health information well enough to know what to do) were not fully invariant across education and organisation, while Scale 8 (Ability to find good health information) was not fully invariant across education, home language and organisation. The follow-up alignment analysis indicated that all non-invariance detectable by this method was, however, metric (non-invariance of factor loadings) rather than scalar. A recent simulation study has shown that scalar non-invariance is a much more important source of bias than metric non-invariance when composite scale scores are compared across groups by, for example, ANOVA and ‘t’ tests [32].

From the perspective of the causal interpretation of the factor model, factor loadings are interpretable as validity coefficients [32, 39] in that they represent the “direct structural relation” between the latent variable and the indicator ([39], p. 197). Thus non-invariance of factor loadings reflects variation in the validity of the item as a measure of the latent construct in particular population sub-groups. In most instances this variation in HLQ item validity is readily interpretable. Thus, for example, the item ‘Get health information … you understand’ was found to have a higher factor loading in the group where English was not typically spoken at home, suggesting that ‘understanding’ health information has enhanced validity as an indicator of the ‘Ability to find good health information’ compared with the other factor indicators for this specific population group. Similarly, two items (‘Ask healthcare providers questions …’; and ‘Work out what the best care is …’) had comparatively enhanced validity for respondents from municipal community services whose clients may, generally, have had less familiarity and ease engaging with health practitioners, while both items that referred to understanding information delivered by healthcare providers had lower validity for clients of a domiciliary nursing service where regular contact with a specific provider may have reduced the salience of these items in comparison with the other items in the scale that referred to written health information. Such variations in item validity will be an important consideration for group comparisons if items are weighted in relation to their factor loadings in the generation of composite scores, but will be of limited concern if items are equally weighted as is typical with HLQ scoring.

When researchers, program managers and policymakers wish to make decisions on services or program needs of specific groups from data obtained from questionnaires, measurement invariance, particularly scalar invariance, is critically important. A questionnaire that is invariant returns unbiased estimates of mean differences or similarities of groups and unbiased estimates of other associations with exogenous variables. This study has demonstrated that comparisons across the great majority of population subgroups were invariant, and when non-invariant were very likely to involve factor loadings rather than the more critical item intercepts suggesting unbiased estimates of health literacy differences using composite scores can be obtained to support program and policy decisions.

### Limitations

While this study sought to provide evidence to support the valid use of the HLQ in the community-setting, it was limited to the use of the English-language HLQ and to data provided by clients of 8 organisations in one state in Australia. Additionally, while care was taken to select organisations from regions in the state with diverse sociodemographic and geographic characteristics, the healthcare organisations studied were in a sense self-selected in that they all responded positively to invitations to participate. Furthermore, while the organisations recruited for the study were encouraged to collect HLQ data from a sample that was as representative as possible of their target group, with substantial efforts to collect data from the ‘harder-to-reach’ clients, these efforts may not have been fully successful. These study characteristics potentially restrict the generalisability of the results and should be kept in mind by organisations in other regions in Australia and other English-speaking countries who intend to use the HLQ to study their client intakes. In particular, the study may have under-represented respondents with lower health literacy. It is arguable that such under-representation might influence the positive findings of measurement invariance across variables such as respondent education and home language. Accumulated experience with the use of the HLQ with difficult-to-reach client groups should assist in addressing this issue in the future.

Similarly, the present results may not be directly applicable to the use of the HLQ in other languages and cultures. Validity studies of translations of the HLQ are underway in German, Dutch, Czech, French, Spanish and other languages or, for the Danish version, are recently published [40], thus the process of cross-cultural validation for the use, interpretations and recommendations for action derived from the questionnaire are underway in these other settings.

Additionally, the present study does not address the issue of the sensitivity of the HLQ to change anticipated to derive from health-literacy focussed interventions, nor does it address whether any observed change is reliable and clinically meaningful. Sensitivity studies of this kind require longitudinal data, at least baseline to follow-up, and should include preliminary investigation of the longitudinal invariance of the scales.

Finally, the length of the HLQ might be seen as a limitation when used in some settings such as clinical locations. While the HLQ itself consists of 44 items and is typically accompanied by 13 or more sociodemographic and health status questions it was successfully administered in the present study by busy clinicians in the course of their usual clinical work. This suggests that the HLQ is written using words and concepts that respondents find straightforward to understand and can answer quite quickly. As there are 9 scales in the HLQ, the 44 items are necessary to ensure scale reliability while maintaining comprehensive coverage of the multidimensional health literacy concept. While comprehensive coverage of the health literacy construct is required in many studies, some, such as national surveys [41] and studies seeking to answer questions about select aspects of health, may use only one or more of the scales.

## Conclusion

The HLQ is currently being used in a wide range of settings across over 30 countries in over 15 languages and this number is growing rapidly. While health literacy is receiving widespread application in population surveys [42], health promotion settings [43] and in supporting equitable approaches to health service improvement [44] it is insufficient for users to be motivated to use the constructs and scales because of their face value. In addressing the principal goal set for this paper we have provided a rigorous examination of the psychometric properties of the HLQ when used across a diverse group of community-based healthcare agencies. The paper thus provides researchers, program managers and policymakers with a rich range of robust evidence by which they can make judgements about the appropriate use of the HLQ for their setting.

## Abbreviations

ANOVA: Analysis of Variance
AVE: Average variance extracted
BSEM: Bayesian structural equation modelling
CFA: Confirmatory factor analysis
CI: Confidence interval
HACC: Home and Community Care services
HARP: Hospital Admission and Risk program
HLQ: Health Literacy Questionnaire
HREC: Human Research Ethics Committee
ICM: Independent clusters measurement model
MCMC: Monte Carlo Markov Chain
ML: Maximum likelihood
MLR: Robust maximum likelihood estimation
Ophelia: Optimising Health Literacy and Access project
PPP: Posterior Predictive Probability value
SEM: Structural equation modelling
WHO: World Health Organisation
WLSMV: Weighted least-squares estimation
